# Awareness and knowledge of female genital schistosomiasis in a population with high endemicity: a cross-sectional study in Madagascar

**DOI:** 10.3389/fmicb.2023.1278974

**Published:** 2023-10-09

**Authors:** Pia Rausche, Rivo Andry Rakotoarivelo, Raphael Rakotozandrindrainy, Rivo Solotiana Rakotomalala, Sonya Ratefiarisoa, Tahinamandranto Rasamoelina, Jean-Marc Kutz, Anna Jaeger, Yannick Hoeppner, Eva Lorenz, Jürgen May, Dewi Ismajani Puradiredja, Daniela Fusco

**Affiliations:** ^1^Department of Infectious Disease Epidemiology, Bernhard Nocht Institute for Tropical Medicine, Hamburg, Germany; ^2^German Center for Infection Research, Hamburg-Borstel-Lübeck-Riems, Hamburg, Germany; ^3^University Fianarantsoa, Fianarantsoa, Madagascar; ^4^University Antananarivo, Antananarivo, Madagascar; ^5^Centre Hospitalier Universitaire Androva, Mahajanga, Madagascar; ^6^Centre Infectiologie Charles Mérieux, Antananarivo, Madagascar; ^7^Department of Tropical Medicine I, University Medical Center Hamburg-Eppendorf (UKE), Hamburg, Germany

**Keywords:** female genital schistosomiasis, women’s health, public health, unmet medical needs, awareness, knowledge

## Abstract

**Introduction:**

Female genital schistosomiasis (FGS) is a neglected disease with long-term physical and psychosocial consequences, affecting approximately 50 million women worldwide and generally representing an unmet medical need on a global scale. FGS is the chronic manifestation of a persistent infection with *Schistosoma haematobium*. FGS services are not routinely offered in endemic settings with a small percentage of women at risk receiving adequate care. Madagascar has over 60% prevalence of FGS and no guidelines for the management of the disease. This study aimed to determine FGS knowledge among women and health care workers (HCWs) in a highly endemic area of Madagascar.

**Methods:**

A convenience sampling strategy was used for this cross-sectional study. Descriptive statistics including proportions and 95% confidence intervals (CI) were calculated, reporting socio-demographic characteristics of the population. Knowledge sources were evaluated descriptively. Binary Poisson regression with robust standard errors was performed; crude (CPR) and adjusted prevalence ratio (APR) with 95% CIs were calculated.

**Results:**

A total of 783 participants were included in the study. Among women, 11.3% (*n* = 78) were aware of FGS while among the HCWs 53.8% (*n* = 50) were aware of FGS. The highest level of knowledge was observed among women in an urban setting [24%, (*n* = 31)] and among those with a university education/vocational training [23% (*n* = 13)]. A lower APR of FGS knowledge was observed in peri-urban [APR 0.25 (95% CI: 0.15; 0.45)] and rural [APR 0.37 (95% CI 0.22; 0.63)] settings in comparison to the urban setting. Most HCWs reported other HCWs [40% (*n* = 20)] while women mainly reported their family [32% (*n* = 25)] as being their main source of information in the 6 months prior to the survey.

**Discussion and conclusions:**

Our study shows limited awareness and knowledge of FGS among population groups in the highly endemic Boeny region of Madagascar. With this study we contribute to identifying an important health gap in Madagascar, which relates to a disease that can silently affect millions of women worldwide. In alignment with the targets of the NTD roadmap, addressing schistosomiasis requires a paradigm shift for its control and management including a greater focus on chronic forms of the disease.

## Introduction

Awareness and knowledge of diseases are key elements for their prevention, early detection and successful management (Aerts et al., 2020). Neglected tropical diseases (NTDs) are a group of diseases suffering, more than others, from lack of awareness and knowledge among both health policy makers as well as health care users and providers (Ortu and Williams, 2017).

Lack of awareness and knowledge about these diseases among stakeholders can contribute to the emergence of unmet medical needs (UMN) (Prusty, 2014; Merki-Feld et al., 2018; Vreman et al., 2019). UMN can be generally defined as conditions without satisfactory diagnostics, prevention or treatment (Vreman et al., 2019). While UMN are a highly debated public health issue (Zhang et al., 2021), it is well recognized that they ultimately contribute to the increasing burden of diseases (Chen et al., 2022), particularly concerning those that are rare (As-Sanie et al., 2019) or less prominent in the global medical sphere, such as NTDs (Chami and Bundy, 2019). Yet, these conditions could potentially be effectively managed with minimal investments.

Human schistosomiasis is a vector-borne NTD with a zoonotic life cycle that occurs primarily in tropical areas. Schistosomiasis prevalence is particularly high in sub-Saharan Africa (SSA) with more than 80% of the global disease burden (Boissier et al., 2016; McManus et al., 2018). Schistosomiasis is caused by six different species of the trematode schistosome, of which *Schistosoma mansoni* and *Schistosoma haematobium* are the most frequent worldwide (Gryseels et al., 2006). The disease leads to chronic inflammations induced by the deposition of *Schistosoma* eggs that can calcify and lead to major detrimental health outcomes (McManus et al., 2018).

The NTD roadmap, released by the World Health Organization (WHO) in 2021 (World Health Organization, 2021), targets the elimination of the disease as a public health problem by 2030 in all endemic countries. Progress on control has been made through vertical programs based on mass drug administration (MDA) with praziquantel, used as preventive chemoprophylaxis in school aged children. These strategies systematically neglected adults, favouring the conditions for the development of chronic forms of the disease especially in highly endemic contexts (Kura et al., 2020; Gruninger et al., 2023). Chronic intestinal schistosomiasis caused by *S. mansoni* can lead to hepato-splenomegaly and portal hypertension while chronic urogenital schistosomiasis caused by *S. haematobium* increases the risk of developing squamous bladder cancer and can lead to male and female genital schistosomiasis.

Female genital schistosomiasis (FGS) is the chronic manifestation of schistosomiasis induced by a persistent infection with *S. haematobium* through the deposition and calcification of eggs in the female genital tract (Mazigo et al., 2022a). The global burden of FGS is unknown most likely due to under-reporting (Engels et al., 2020), but it is estimated that it affects 40 to 56 million women and girls worldwide (Hotez et al., 2019a). On the basis of current available data, between 33% and 75% of women and girls suffer from infection with *S. haematobium* and are at risk of developing FGS (Hotez and Whitham, 2014). Furthermore, FGS has been shown to increase the risk of HIV infection, and is suspected to play a role in the onset and/or progression of cervical cancer (Hotez et al., 2019a,b; Patel et al., 2021).

FGS induces cervical lesions and can lead to ectopic pregnancies and infertility (Kjetland et al., 2012). Moreover, it has been linked to psychosocial consequences, such as stigma and depression as well as a loss of work productivity and therefore reduced income (Hotez and Whitham, 2014). The latter contributes to the perpetuation of the vicious cycle of poverty frequently associated with NTDs (de Rijk et al., 2021). FGS shares common clinical features with sexually transmitted diseases (STDs) such as pain, itching or vaginal discharge (Mazigo et al., 2021) frequently leading to misdiagnosis, inappropriate treatment, social stigma, and further risk of under detection and underreporting. Limited awareness and treatment of FGS has been described both among HCWs and the general population in several endemic countries such as Ghana, Tanzania, and Cameroon (Kukula et al., 2019; Masong et al., 2021; Mazigo et al., 2021, 2022a).

Standard treatment for FGS is a single dose administration of 40 mg/kg of praziquantel (McManus et al., 2018). However, mounting evidence indicates limitations of this treatment, particularly concerning its effectiveness in resolving the typical lesions caused by the disease (Norseth et al., 2014). The diagnosis of FGS is particularly challenging since the condition can be present also without living parasites actively excreting eggs. Thus, in the absence of FGS specific biomarkers, microscopy is not suitable for its diagnosis (Kjetland et al., 2014; WHO, 2015). Instead colposcopy through visual inspection of characteristic lesions, such as yellow sandy patches, abnormal blood vessels and rubbery papules, is designated as the standard diagnostic. Colposcopy is a relatively complex procedure (Norseth et al., 2014) that requires trained staff as well as appropriately equipped facilities (WHO, 2015). In resource limited settings (Mazigo et al., 2022b) these facilities are often not widely available and primarily concentrated at the tertiary levels of care (Xue et al., 2020). Nonetheless, the global lack of awareness surrounding FGS (Conseil National du Recensment de la Population et de l’habitation, 2021; Rasoamanamihaja et al., 2023) adds complexity to its identification, even in high-income settings where infrastructure and professional capacity are typically not constraints (Rasoamanamihaja et al., 2023). Consequently, FGS diagnosis and treatment represents an UMN in both endemic and non-endemic contexts.

Madagascar is one of the countries with the highest prevalence of schistosome infections worldwide (Gruninger et al., 2023) with recent data showing also high prevalence of FGS (over 60%) (Kutz et al., 2023). In the country, there are no guidelines, which specifically pertain to the management of FGS (Rasoamanamihaja et al., 2023). The underlying assumption of our study is that FGS policies, guidelines and programs in Madagascar require (among others) awareness and knowledge of the disease. However, little is known about FGS awareness and knowledge among both women and HCWs in Madagascar. Our study aimed to address this gap by determining FGS knowledge among these two population groups in the highly *S. haematobium* endemic rural region of Boeny in Madagascar (Gruninger et al., 2023).

## Methods

### Study design, area and population

This cross-sectional survey study was conducted in the rural region of Boeny in Madagascar. Data were collected using a structured questionnaire administered by trained interviewers. The Boeny region has an estimated population of 543,200 inhabitants. Four municipalities within the region have been selected for the implementation of the study: Mahajanga (−15° 42′ 59.99″S-46°18′ 60.00″ E), Antanambao Andranolava (15° 57′ 59.99″S-46° 40′ 59.99″ E), Maravoay (−16° 06′ 38.30″ S-46° 38′ 37.79″ E) and Ankazomborona (−16° 06′ 60.00″S-46° 44′ 59.99″ E). The city of Mahajanga can be described as urban with 87,660 inhabitants (Conseil National du Recensment de la Population et de l’habitation, 2021), while the town of Marovoay with its 34,000 inhabitants can be classified as peri-urban. The two remaining study sites consist of the ten communities of Ankazomborona and eight communities of Antanambao-Andranolava with, respectively, 23,000 and 3,000 inhabitants corresponding to rural characteristics (INSTAT Madagascar - Institut National de la Statistique, 2022). The study sites have been selected according to variations in urbanicity, and the overall high estimated FGS prevalence of more than 60% in the region (Kutz et al., 2023).

### Participant sampling, recruitment and eligibility criteria

Adult women from among the general population as well as male and female HCWs were selected using a convenience sampling approach. The total sample size was rounded to 1,000 as described in Conroy (2018), though, due to the COVID-19 pandemic, a smaller sample size was reached and a total of 820 individuals were surveyed. Participants were approached at markets, schools, health facilities, and their homes to assess their FGS awareness and knowledge. All participants were asked to sign an informed consent. In case of illiteracy an impartial witness was involved.

Inclusion criteria of the study were: (i) healthcare professionals working at the primary health care centres or female community members of Boney at markets, schools, health facilities or their home, (ii) older than 18 years of age, (iii) fluent in French and/or Malagasy, and (iv) willing and able to provide written informed consent.

### Data collection and data management

Data were collected between 24/08/2020 and 04/09/2020. A paper-based questionnaire was administered and answered in either Malagasy or French. The questionnaire was structured into three thematic sections: (a) socioeconomic information, (b) awareness and knowledge of FGS, (c) health-seeking behaviour for non-medical personnel, and FGS treatment and diagnostic knowledge for HCWs. All questions were asked in a non-prompted format and if none of the pre-specified categories were applicable, additional answers were recorded in a free text/open response format.

All study participants were assigned a unique patient identifier (PID) to ensure data protection and pseudonymization. Questionnaires underwent a quality check following standard operating procedures. Double data entry was performed using the REDCap electronic data capture tools hosted at the Bernhard Nocht Institute for Tropical Medicine, Hamburg, Germany (Harris et al., 2009, 2019). Quality control of data processing and data validation was undertaken at regular intervals by data quality managers. The dataset was screened for missing values. Missing data corresponding with the exclusion criteria led to the exclusion of participants from the study. Missing values not leading to study exclusion were descriptively reported.

### Statistical analysis

Descriptive statistics were calculated to summarize the socio-demographic characteristics of the population stratified by women and HCWs. Awareness of FGS was described through proportions and 95% confidence intervals (CI). The presence of awareness is assumed if a participant states that they have heard of FGS before today.

An FGS knowledge score was computed including five domains: (i) symptoms, (ii) transmission pathways, (iii) protective measures, (iv) consequences of the disease and (v) contribution of individuals to transmission. A maximum of 50 points was assigned to each participant, divided into 10 maximum points per domain. Both correct and incorrect answers were scored for each domain. Points were awarded for both answers mentioned correctly and unmentioned if correct. If no answers were ticked at all, 0 points were assigned. Open text answers were assigned to existing categories if possible. Score calculation is displayed in Supplementary Table S1. The overall score ranged from 0 to 50 points with the following categories: 0–20: no knowledge, 21–30: low knowledge, 31–40: medium knowledge, 41–50: high knowledge. Afterwards, sources of information were described through proportions for women and HCWs aware of FGS.

To calculate crude and adjusted prevalence ratios (CPR and APR) with 95% confidence intervals, a binary Poisson regression with robust standard errors was performed (Barros and Hirakata, 2003). FGS awareness was considered as dependent variable and age group, urbanization of recruitment, place of interview, education and occupation as independent variables. The regression analysis was performed exclusively for female community members due to a limited sample size in the HCWs population. All statistics were performed using R version 4.2.2 (R Foundation for Statistical Computing, Vienna, Austria).

### Ethical considerations

This study was approved by the Ethics Committee Hamburg State Medical Chamber (protocol number PV7309) and the National Ethics Committee of Madagascar (protocol number 052/MSANP/SG/AGMED/CERBM). No person who met the inclusion criteria was excluded as a participant in this study based on their sexual orientation, gender identity, political, ethnic affiliation, or socioeconomic position. Participants had the right to refuse to participate and to withdraw informed consent at any time without giving reasons. No financial incentives were given for study participation.

## Results

### Study population

A total of 93 HCWs and 727 women were surveyed. During the data cleaning process, 33 women were excluded due to the exclusion criterion of age less than 18 years. Missing age also resulted in exclusion from the study of 4 individuals. The final study population included in the analysis was then 93 HCWs and 690 women (Table 1).

**Table 1:** Socio-demographic characteristics of surveyed women and HCWs in the Boeny region of Madagascar.

	Women	Healthcare workers
	*n* (%)	*n* (%)
Total	690 (100)	93 (100)
**Language of interview**		
Malagasy	690 (100)	91(97.8)
French	0 (0)	2 (2.2)
**Setting** ^a^		
Healthcare facility	116 (16.8)	91 (97.8)
Community	574 (83.2)	2 (2.2)
**Age group**		
18–25	247 (35.8)	27 (29.0)
26–35	224 (32.5)	29 (31.2)
36–45	117 (17.0)	23 (24.7)
46+	102 (14.8)	14 (15.1)
**Urbanization**		
Urban	129 (18.7)	54 (58.1)
Peri-urban	284 (41.2)	12 (11.2)
Rural	277 (40.2)	27 (25.1)
**Education** ^b^		
Primary school and less	281 (40.7)	0 (0.0)
Secondary education	353 (51.2)	4 (4.3)
University/vocational training	56 (8.1)	88 (94.6)
**Occupation** ^c^		
Non-farmer/-fisher	261 (37.8)	N/A
Farmer/-fisher	429 (62.2)	N/A
**Religion** ^d^		
No religion	91 (13.2)	0 (0.0)
Christian	505 (67.3)	84 (90.3)
Muslim	24 (3.5)	6 (6.5)
Other	70 (10.1)	3 (3.3)

a Healthcare facilities are hospitals or primary health care centres (CSB), community is including markets, participants homes and other public places.

b “Primary school and less” includes no education; NA = 1 for HCW.

c Non-farmer/-fisher includes no occupation, employee, business, housewives, students and others (no answer, tailor, assistant or server).

d Other do include: do not know, no answer, divers sects.

The interviews were mostly conducted in Malagasy with the total of women surveyed in Malagasy, and just the 2.2% (*n* = 2) of the HCWs in French. Most common location of interviews for HCWs were, by design, health care facilities where 97.8% (*n* = 91) of HCWs were interviewed. In contrast, 83.2% (*n* = 574) of women were recruited in community settings such as participants homes or markets and 16.8% (*n* = 116) at health care facilities. The distribution of urban, peri-urban and rural study sites differed between women and HCWs. While among the women, 18.7% (*n* = 129) were surveyed in an urban setting, 41.2% (*n* = 284) were surveyed in a peri-urban and 40.2% (*n* = 277) in a rural setting, HCWs were most commonly interviewed in an urban setting [58.1% (*n* = 54)]. A total of 11.2% (*n* = 12) and 25.1% (*n* = 27) HCWs were interviewed in peri-urban and rural sites, respectively. The most represented age group among women was 18–25 years while among HCWs was 26–35 years.

The lowest level of education among HCWs was secondary education, reported for 4.3% (*n* = 4) of the population, while primary education or less among women was reported from 40.7% (*n* = 281) of the population. While 51.2% (*n* = 352) of the women indicated a secondary education, respectively 8.1% (*n* = 56) of the women and 94.6% (*n* = 88) of the HCWs had a university degree or completed vocational training.

Among women, 62.2% (*n* = 429) were farmers or fishers. Specific occupation was not recorded for HCWs. The majority of participants in both groups identified as Christian with 67.3% (*n* = 505) of the women and 90.3% (*n* = 84) of the HCWs. While none of the HCWs said they have no religion, 13.2% (*n* = 91) participants among the women indicated no religion.

### Awareness of FGS in the study population

**Figure 1:**
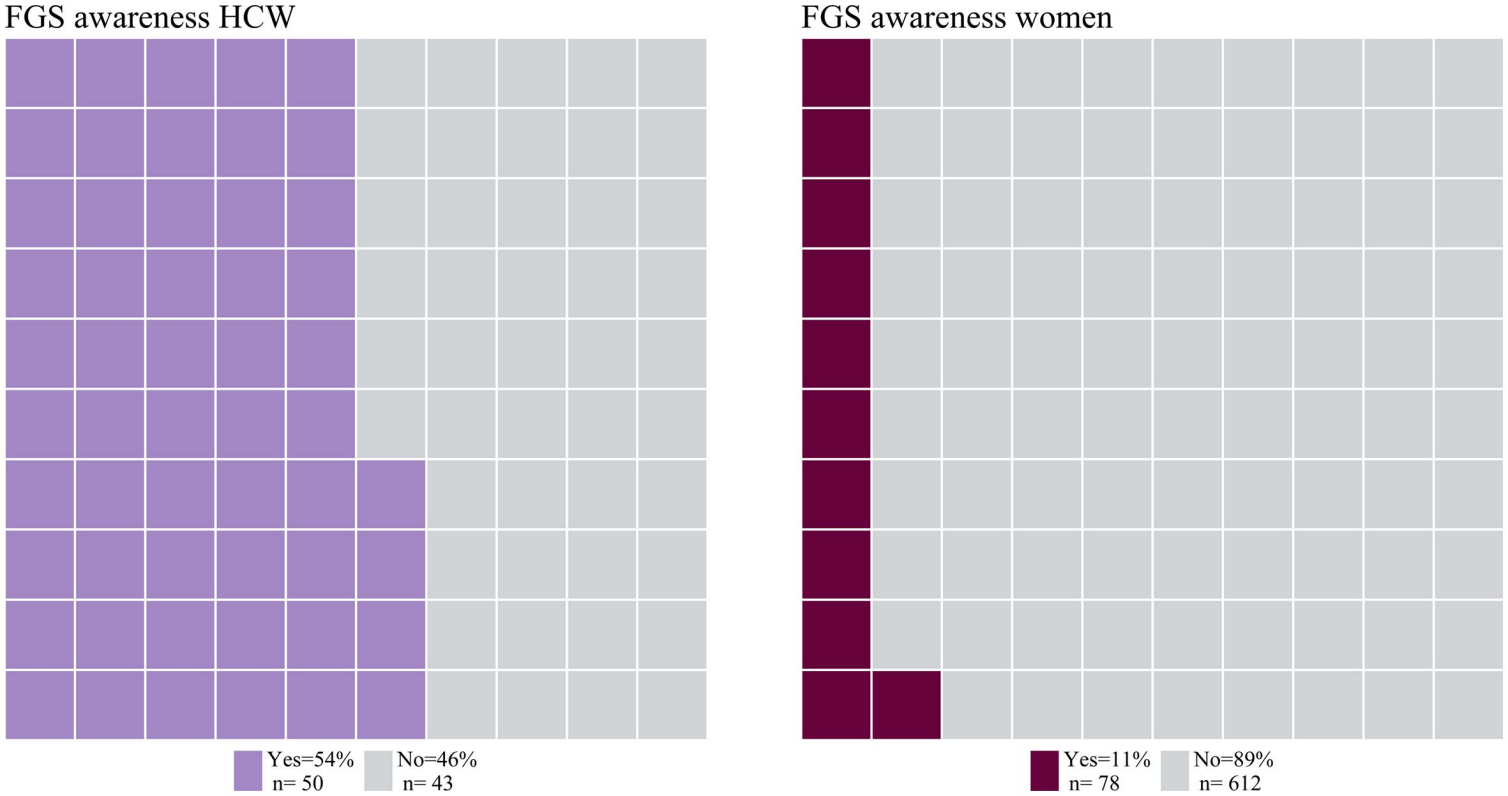
Awareness of FGS among HCW and women. Proportional waffle plot for *n* = 93 HCW and *n* = 690 women, one square representing 1% of the participants in this group.

Among women, 11.3% [*n* = 78 (95% CI: 9.0–13.9)] were aware of FGS, compared to the 53.8% [*n* = 50 (95% CI 43.1–64.2)] of the HCWs (Figure 1). The 15.2% [*n* = 34 (95% CI: 10.7–20.6)] and 12.8% [*n* = 15 95% CI: (7.4–20.3)] of women in the age groups 26–35 and 36–45, respectively, were aware of FGS, while lower proportions of FGS awareness were observed in the age groups 18–25 [9.3%, *n* = 23 (95% CI: 6.0–13.6)] and 46+ [5.9%, *n* = 6 (95% CI: 2.2–12.4)]. The general awareness of FGS among women and HCWs are described in Supplementary Table S2.

Women living in urban areas had a higher awareness of FGS [24.0%; *n* = 31 (95% CI: 16.9–32.3)] as compared to women living in peri-urban or rural areas with 6.7% [*n* = 19 (4.1–10.3)] and 10.1% [*n* = 28 (95% CI, 6.8–14.3)] of awareness, respectively. Similarly, HCWs from the urban study site showed the highest level of general FGS awareness of FGS with 68.5% [*n* = 37 (54.4–80.5%)], while in the peri-urban and the rural locations lower proportions of the study population reported to be aware of FGS with 37.0% [*n* = 10 (19.4–57.6)] and 25.0% [*n* = 3 (5.5–57.2)] respectively.

A small difference in FGS awareness could be observed across occupations. While 13.8% [*n* = 36 (9.9–18.6)] of women not working as farmer or fisher were aware of FGS, only 9.8% [*n* = 42 (7.1–13.0)] of those working as a farmer or fisher showed awareness for the disease.

### Knowledge score

Overall, it can be observed that among the 690 women, 9.3% [*n* = 64 (95% CI 7.2–11.7)] reached low knowledge scores, 1.9% [*n* = 13 (95% CI 1.0–3.2)] medium and 88.8% [*n* = 613 (95% CI 86.3–91.1)] had no knowledge. In total 47.3% [*n* = 44 (95% CI 36.9–57.9)] of HCWs had low knowledge of FGS, 6.5% [*n* = 6 (95% CI 2.4–13.5)] had medium knowledge and 46.2% [*n* = 43(95% CI 35.8–56.9)] had no knowledge of FGS. Neither the women nor the HCWs included in this study reached a high knowledge score.

**Figure 2:**
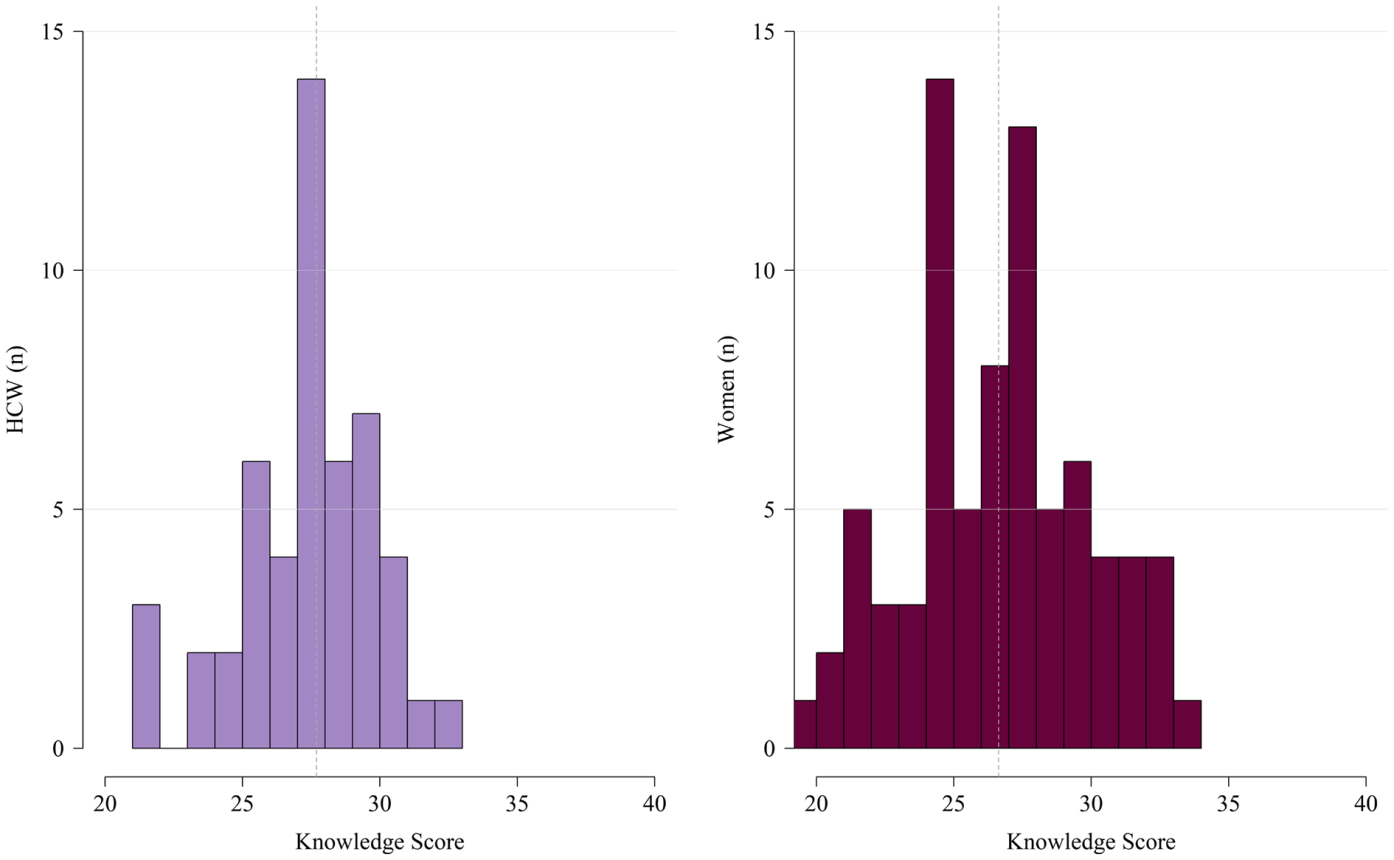
Distribution of knowledge score among HCWs (*n* = 50) and women (*n* = 78) with previous awareness of FGS. Dashed line is representing the median.

The knowledge score for participants aware of FGS prior to the survey is displayed in Figure 2. While HCWs had a slightly higher median score of 28 (IQR: 25–29) with a minimum score of 21 and a maximum score of 32, women had a higher maximum score with a median knowledge score of 27 (IQR: 26–29) with a minimum score of 20 and a maximum score of 34.

### Sources of information

**Figure 3:**
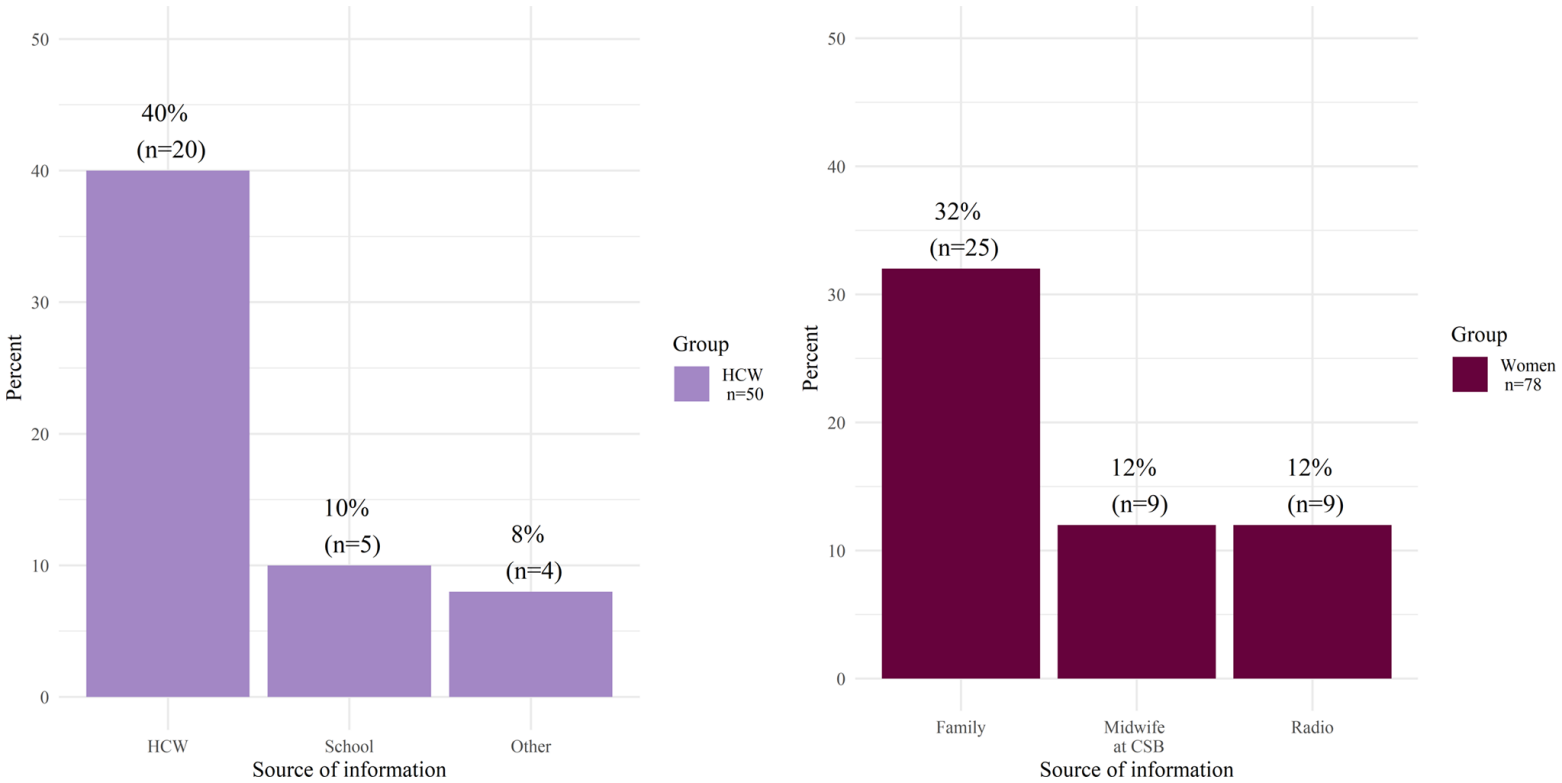
Three most common sources of FGS information 6 months prior to the survey for HCWs, and for women.

Among the 76 women and 50 HCWs who reported awareness of FGS, the source of information about the disease in the last 6 months was investigated and summarised in Figure 3. Family [32.1% (*n* = 25)] as well as midwives and the radio with each 11.5% (*n* = 9) represent the most common sources of information for women.

Information about FGS among HCWs is mostly acquired through other HCWs [40% (*n* = 20)]. School curricula were mentioned by 10% (*n* = 5) of the HCWs as their source of information. Among the HCWs (*n* = 50) and women (*n* = 76) aware of FGS, 36% (*n* = 18) and 26.9% (*n* = 21) respectively mentioned that they did not receive any information in the 6 months prior to the survey. Other less frequently named sources of information were awareness campaigns or community workers for the women, and television for the HCWs.

### Prevalence ratio for FGS awareness among women

CPR and APR were estimated for associations with variables considered as possible influencing factors for FGS awareness. The model was performed exclusively among the women (*n* = 690) because of the limited sample size (*n* = 93) of HCWs. CPR and APR for FGS awareness among women are listed in Supplementary Table S3.

**Figure 4:**
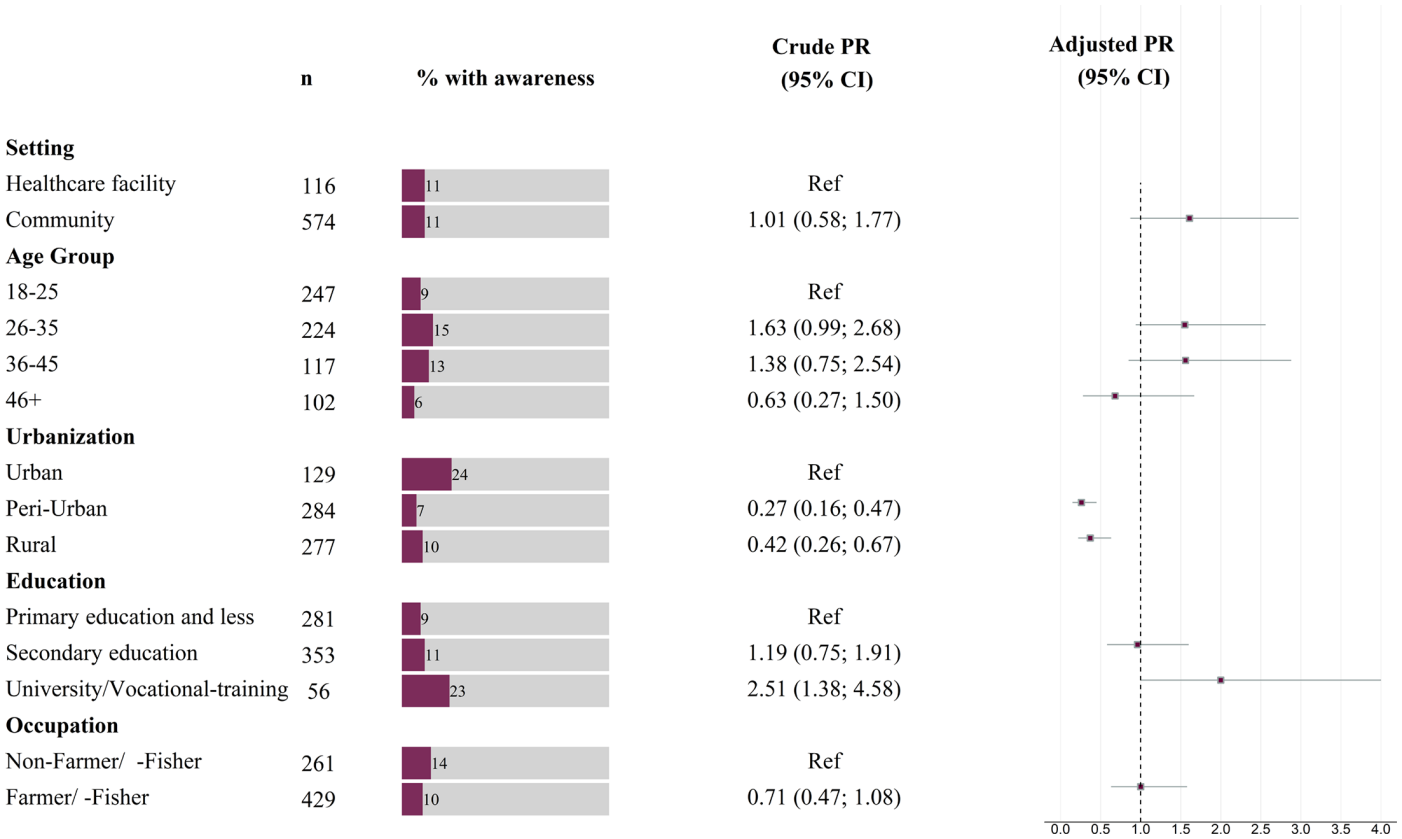
Prevalence ratios for FGS awareness among women (*n* = 690), based on binary Poisson regression models with robust standard errors. Box-whisker plot indicates adjusted prevalence ratio and 95% confidence interval.

The adjusted model displayed in Figure 4 shows a non-significant residual deviance and a moderate variance inflation factor ranging from 1.1–1.4 for all predictors.

Peri-urban locations [APR 0.26 (95% CI: 0.15; 0.45)] and rural locations [APR 0.37 (95% CI 0.22; 0.63)] had shown a reduced prevalence, statistically significant, of 74% and 63% FGS awareness respectively, when compared to the urban location of Mahajanga.

Women who attended university or completed a vocational training had twice the prevalence, statistically significant, of FGS awareness when compared to the group with a primary education or less [APR 2.0 (95% CI 1.00; 4.00)]. However, no difference was found when comparing the prevalence of FGS knowledge among women with secondary education and primary education or less [APR 0.96 (95% CI 0.58; 1.60)].

An increased prevalence of FGS awareness was observed when comparing the location of the interview in a community setting to a location of the interview in a hospital setting [APR 1.61 (95% CI 0.87; 2.97)]. Furthermore, an increased APR was observed for the age groups 26–35 [APR 1.55 (95% CI 0.94; 2.56)], 36–45 [APR 1.56 (95% CI 0.85; 2.88)] and 46+ [APR 0.68 (95% CI 0.28;1.67)] when compared to the age group 18–25. No difference in the prevalence of awareness between farmer/-fisher and non-farmer/-fisher was found [APR 1.00 (95% CI 0.63, 1.58)].

## Discussion

Limited FGS awareness and knowledge can result in UMN related to FGS. Our study shows overall low awareness and knowledge of the disease in the highly endemic context of the Boeny region in Madagascar, and contributes to identifying an important health gap, which relates to a disease that can silently affect millions of women worldwide.

The main findings of our study show that 54% of HCWs and 11% of women were aware of FGS while both HCWs and women have limited knowledge of the diseases according to the score established and computed in this study. We report that living in an urban area [non-urban settings: APR 0.26 (95% CI: 0.15; 0.45); APR 0.37 (95% CI 0.22; 0.63)] and having completed university education or vocational training [APR 2.0 (95% CI 1.00; 4.00)] are factors influencing FGS awareness in women. Interestingly, we observe that those working as farmers, commonly reported to be at more at risk for schistosomiasis (Gruninger et al., 2023), are slightly less aware of FGS even though, from the model, we cannot conclude that this factor directly influences awareness. Finally, we report that in both groups, the most common sources of FGS knowledge were peers: other HCWs among HCWs and family members among women. Our main findings are aligned with existing literature from other endemic countries (Kukula et al., 2019; Mazigo et al., 2021, 2022b) and with an exploratory and qualitative study conducted in Madagascar in 2022, on 76 women in the Ambanja district (Schuster et al., 2022).

Schistosomiasis is a high burden disease particularly in SSA countries, which is mostly being addressed with MDA programs (French et al., 2018). This has generated a general awareness and knowledge of the disease among policy makers, HCWs, the at-risk populations and other stakeholders (Christinet et al., 2016). MDAs have been promoted and implemented with the intent of controlling the disease by decreasing its transmission in highly endemic areas (Kokaliaris et al., 2022). Unfortunately, since this strategy has not shown the expected public health impact so far (Li et al., 2019) there is the distinct possibility of an increase in chronic forms of the disease, which remain mostly undetected and untreated (Christinet et al., 2016). Our findings show that in a context that is highly endemic for FGS (Kutz et al., 2023), awareness and knowledge of the disease among both users and providers of health services are low. This may be one important reason for the notable lack of both supply and demand of health services for the detection and management of the FGS in the country (Rasoamanamihaja et al., 2023). Neglecting chronic forms of schistosomiasis, such as FGS, not only contributes to an increasing disability-adjusted life years (DALYs), decreasing quality adjusted life years (QALYs), and perpetuating the vicious cycle of poverty for affected populations, but also to the transmission of the infection in highly endemic areas. The 2021–2030 WHO NTD roadmap (World Health Organization, 2021) addresses the elimination of schistosomiasis as a public health problem by 2030. If immediate actions to address the chronic forms of the disease are not taken, the WHO target will be hard to reach. Chronic forms of schistosomiasis require a paradigm shift in the management of the disease, moving away from conventional mass strategies within vertical programs. Instead, the focus should be on integrated services that can cater more to the needs of affected individuals.

Furthermore, limited awareness and knowledge of FGS can contribute to exacerbate gender gaps and social inequalities among vulnerable populations. FGS presents signs and symptoms that can be similar to/misinterpreted as being sexually transmitted diseases (STDs) (Mazigo et al., 2022b). Despite the progress made in the control and management of STDs worldwide (Zheng et al., 2022), social stigma still remains one of the major problems associated with these diseases (Lee and Cody, 2020; Mazigo et al., 2022b). The major consequence of social stigma is the fear of the population, especially among women, of using health services (Rusch et al., 2008) due to the consequent marginalisation that they could experience in their communities (Avuvika et al., 2017). Additionally, a lack of knowledge of the FGS among HCWs can lead to incorrect diagnosis (Mazigo et al., 2022b) and treatment (WHO, 2015). Raising awareness and knowledge about FGS, could help encourage women to seek timely medical care, prevent long term consequences, such as infertility or cancer associated with the disease (Mazigo et al., 2022a), and reduce antimicrobial resistance due to inappropriate use of antibiotics (Nemungadi et al., 2022).

Notably, our data show that both HCWs and women in the general population report peers as being their main source of information about the disease. Peer communication among patients has proven to be effective in increasing awareness and knowledge of different diseases ranging from HIV (Ayala et al., 2021) to cancer (Ancker et al., 2009), and COVID-19 (Shoghli et al., 2023). Various health strategies have already been conceptualised, implemented and frequently integrated at different levels of care depending on the type of disease and context (Ancker et al., 2009; Markowski et al., 2021). For instance, peer leaders in the fight against HIV have been involved in several health programs in highly endemic countries, such as South Africa (Ayala et al., 2021), not only to raise awareness about the disease but also to promote health services, such as prevention and screening in rural communities (Mannoh et al., 2022). Similarly, for FGS, the involvement of community leaders in health communication campaigns could improve health seeking behaviour of the affected populations. Unfortunately, in the absence of an easy, field applicable diagnostic, FGS community-based screening programs must rely on highly equipped health facilities, which makes the design and implementation of widely accessible and sustainable services challenging (Xue et al., 2020).

Additionally, our findings show that those with higher education (APR 2.0 [95% CI 1.00; 4.00]) and those living in urban settings [non-urban settings: APR 0.26 (95% CI: 0.15; 0.45); APR 0.37 (95% CI 0.22; 0.63)] have a higher prevalence of FGS awareness. In Madagascar, most of the population lives in remote areas and more than 5 km away from health care facilities (U.S. Agency for International Development, 2022), which represents a major challenge to the provision of access to care and to (health) education (Rasoamanamihaja et al., 2023). In general, FGS is rarely mentioned in medical textbooks nor a topic covered as part of continuing medical education (CME) programs (UNAIDS, 2019). The lack of FGS-related CME in combination with a lack of routine services and thus little exposure of HCWs to direct medical practice for FGS, further complicates the translation of theory into practice (Gupta et al., 2017). In fact, the implementation of CME programs is challenging in Madagascar not only for the common barriers encountered in LMICs (Merry et al., 2023), such as budget limitations or scarcity of equipment (English et al., 2016), but also due to the scarcity of medical personnel in health facilities who cannot be readily replaced in their absence (U.S. Agency for International Development, 2022).

This study provides valuable insights on the under-researched topic of FGS awareness and knowledge among a comparatively large sample of women and HCWs in a highly endemic country. Moreover, it introduces the concept of a score to assess knowledge of the disease, which helps to identify specific gaps among health care users and providers, which need addressing. Finally, its findings provide elements for the design and implementation of FGS-related awareness campaigns and training, including among others the use of peer education. Despites its strengths, our study is not without limitations. Firstly, this study used a convenience sampling approach, which can have implications in terms of the generalizability from the enrolled population to the wider two target populations of both women from the general population and HCWs. Further, our data are drawn from a cross-sectional survey, meaning that there are limitations to the interpretability of risk associations. Since recruitment took in part place at health care facilities, which had been previously part of other schistosomiasis-related research projects, a bias may have been introduced, which means that an overestimation of awareness and knowledge among participants cannot be excluded. Furthermore, the conceptualization of the knowledge score was specifically designed to fit the context of the study. Even though it is informed by other existing tools, ours is not validated. Due to the structure of the data collection tool, we cannot report differences in the level of knowledge segregated by different professions of the health care system (i.e., doctors, nurses, midwives, community health care workers) nor address age among specific groups (i.e., reproductive age). Moreover, the high proportion of Christians within our sample does not allow to make any conclusion about variations across religious groups and possible differences in social norms and behaviours in terms of access to and use of health services (Kang et al., 2020). Finally, in our study we exclusively address women, since they are the ones directly affected by FGS. Further research is needed to account for the role of men in the possible transfer of and access to FGS awareness and knowledge in the communities within this setting.

## Conclusion

In conclusion, our study identifies important gaps in FGS-related knowledge among affected women and healthcare workers in Madagascar. In alignment with the WHO NTD Road Map, this has important implications for the control and management of schistosomiasis, and its more chronic forms. Raising awareness and knowledge of chronic forms of NTDs can help address diseases that can silently affect individuals worldwide.

## Data availability statement

The raw data supporting the conclusions of this article will be made available by the authors, without undue reservation.

## Ethics statement

The studies involving humans were approved by Ethics Committee Hamburg State Medical Chamber (protocol number PV7309) and the National Ethics Committee of Madagascar (protocol number 052/MSANP/SG/AGMED/CERBM). The studies were conducted in accordance with the local legislation and institutional requirements. The participants provided their written informed consent to participate in this study.

## Author contributions

PR: Data curation, Formal analysis, Methodology, Visualization, Writing – original draft. RAR: Conceptualization, Funding acquisition, Project administration, Supervision, Writing – review & editing. RR: Project administration, Supervision, Writing – review & editing. RSR: Supervision, Writing – review & editing, Investigation. SR: Investigation, Supervision, Writing – review & editing, Project administration. TR: Conceptualization, Funding acquisition, Investigation, Project administration, Writing – review & editing. J-MK: Investigation, Project administration, Supervision, Writing – review & editing. AJ: Data curation, Writing – review & editing. YH: Investigation, Writing – review & editing. EL: Conceptualization, Formal analysis, Funding acquisition, Methodology, Supervision, Writing – review & editing. JM: Resources, Writing – review & editing. DP: Conceptualization, Funding acquisition, Methodology, Supervision, Writing – review & editing. DF: Conceptualization, Data curation, Funding acquisition, Investigation, Methodology, Project administration, Supervision, Writing – original draft.
