# Supplementary material for: Awareness and knowledge of female genital schistosomiasis in a population with high endemicity: a cross-sectional study in Madagascar
Source: Front Microbiol. 2023 Oct 9;14:1278974. doi: 10.3389/fmicb.2023.1278974 (PMC10598593; doi:10.3389/fmicb.2023.1278974)
Supplement: Supplementary file 1 [file Table_1.DOCX]

Supplementary Table 1

**Awareness and knowledge of Female Genital Schistosomiasis in a population with high endemicity: a cross-sectional study in Madagascar**

Pia Rausche^1,2^, Rivo Andry Rakotoarivelo^3^, Raphael Rakotozandrindrainy^4^, Rivo Solotiana Rakotomalala^5^, Sonya Ratefiarisoa^5^, Tahinamandranto Rasamoelina^6^, Jean-Marc Kutz^1,2^, Anna Jaeger^1^, Yannick Hoeppner^1^, Eva Lorenz^1,2^, Jürgen May^1,2 ,7^, Dewi Ismajani Puradiredja^1^, Daniela Fusco^1,2 *^

^1^ Department of Infectious Disease Epidemiology, Bernhard Nocht Institute for Tropical Medicine, Hamburg, Germany

^2^ German Center for Infection Research, Hamburg-Borstel-Lübeck-Riems, Germany

^3^ University Fianarantsoa, Fianarantsoa, Madagascar

^4^ University Antananarivo, Antananarivo, Madagascar

^5^ Centre Hospitalier Universitaire Androva, Mahajanga, Madagascar

^6^Centre Infectiologie Charles Mérieux, Antananarivo, Madagascar

^7^ Department of Tropical Medicine I, University Medical Center Hamburg-Eppendorf (UKE), Germany

*** Correspondence:**Daniela Fusco
fusco@bnitm.de

**Supplementary table 1**: Calculation of knowledge score:

| **Category** | **Right Answers** | **Overall answer possibilities** | **Points per right answer ticked and wrong answer not ticked** | **Points per category** |
| --- | --- | --- | --- | --- |
| Symptoms | - Vaginal discharge, Bloody discharge, Bleeding after sex, burning sensation, Pelvic pain, Pain during/ after sex, contact bleeding, Intermenstrual bleeding, Infertility, Painful urination, Frequent urination, Blood in urine, Girls can have symptoms too | 23 | 1/2.3 | 10 |
| Protective measures | Drug treatment for infected, Mass drug administration, Chemical treatment of water body, Protect water body, Don’t swim in river/ lakes, Use well water, More latrines/ improved hygiene | 9 | 1/0.9 | 10 |
| Transmission | Bringing in water, Fishing, Washing dishes/ laundry in river or lake, Bathing swimming in lake, Working in rice fields | 12 | 1/1.2 | 10 |
| Contribution transmission | Infected person urinates in water | 3 | 1/0.3 | 10 |
| Consequences | Infertility, Ectopic pregnancy, Involuntary urination, Genital ulceration, Abdominal cramps, Incapacity to conceive, Difficulty to conceive, Increased risk of HIV | 19 | 1/1.9 | 10 |
| **Total** |  |  |  | **50** |
