# Supplementary material for: Awareness and knowledge of female genital schistosomiasis in a population with high endemicity: a cross-sectional study in Madagascar
Source: Front Microbiol. 2023 Oct 9;14:1278974. doi: 10.3389/fmicb.2023.1278974 (PMC10598593; doi:10.3389/fmicb.2023.1278974)
Supplement: Supplementary file 2 [file Table_2.DOCX]

Supplementary Table 2

**Awareness and knowledge of Female Genital Schistosomiasis in a population with high endemicity: a cross-sectional study in Madagascar**

Pia Rausche^1,2^, Rivo Andry Rakotoarivelo^3^, Raphael Rakotozandrindrainy^4^, Rivo Solotiana Rakotomalala^5^, Sonya Ratefiarisoa^5^, Tahinamandranto Rasamoelina^6^, Jean-Marc Kutz^1,2^, Anna Jaeger^1^, Yannick Hoeppner^1^, Eva Lorenz^1,2^, Jürgen May^1,2 ,7^, Dewi Ismajani Puradiredja^1^, Daniela Fusco^1,2 *^

^1^ Department of Infectious Disease Epidemiology, Bernhard Nocht Institute for Tropical Medicine, Hamburg, Germany

^2^ German Center for Infection Research, Hamburg-Borstel-Lübeck-Riems, Germany

^3^ University Fianarantsoa, Fianarantsoa, Madagascar

^4^ University Antananarivo, Antananarivo, Madagascar

^5^ Centre Hospitalier Universitaire Androva, Mahajanga, Madagascar

^6^Centre Infectiologie Charles Mérieux, Antananarivo, Madagascar

^7^ Department of Tropical Medicine I, University Medical Center Hamburg-Eppendorf (UKE), Germany

*** Correspondence:**Daniela Fusco
fusco@bnitm.de

**Supplementary table 2:** Degree of FGS awareness in percent (95% CI)

|  | **General population** | **Healthcare workers** |
| --- | --- | --- |
|  | **% (95%CI)** | **% (95%CI)** |
| *Overall aware* | **11.3 (9.0-13.9)** | **53.8 (43.1-64.2)** |
| **Location of interview** |  |  |
| Healthcare facility | 11.2 (6.1-18.4) | 53.8 (43.1-65.4) |
| Community | 11.3 (8.8- 14.2) | 50.0 (1.3-98.7) |
| **Age group** |  |  |
| 18-25 | 9.3 (6.0- 13.6) | 63.0 (42.4-80.6) |
| 26-35 | 15.2 (10.7-20.6) | 65.5 (45.7-82.1) |
| 36-45 | 12.8 (7.4-20.3) | 21.7 (7.5-43.7) |
| 46+ | 5.9 (2.2-12.4) | 64.3 (35.1-87.2) |
| **Urbanization** |  |  |
| Urban | 24.0 (16.9- 32.3) | 68.5 (54.4- 80.5) |
| Peri-Urban | 6.7 (4.1- 10.3) | 37.0 (19.4-57.6) |
| Rural | 10.1 (6.8-14.3) | 25.0 (5.5-57.2) |
| **Education^1^** |  |  |
| Primary school and less | 9.3 (6.1- 13.3) | N/A |
| Secondary education | 11.0 (8.0–14.8) | 50.0 (6.8-93.2) |
| University/ Vocational- training | 23.2 (13.0-36.4) | 53.3 (42.4-64.1) |
| **Occupation** |  |  |
| Non- Farmer/ - Fisher | 13.8 (9.9-18.6) | N/A |
| Farmer/ Fisher | 9.8 (7.1-13.0) | N/A |

*Abbreviations: CI = Confidence interval, FGS = Female Genital Schistosomiasis, HCW = Health care workers, NA = No participants in this group, N/A= Not applicable, ^1^ NA= 1 for HCW*
