# Supplementary material for: Awareness and knowledge of female genital schistosomiasis in a population with high endemicity: a cross-sectional study in Madagascar
Source: Front Microbiol. 2023 Oct 9;14:1278974. doi: 10.3389/fmicb.2023.1278974 (PMC10598593; doi:10.3389/fmicb.2023.1278974)
Supplement: Supplementary file 3 [file Table_3.DOCX]

Supplementary Table 3

**Awareness and knowledge of Female Genital Schistosomiasis in a population with high endemicity: a cross-sectional study in Madagascar**

Pia Rausche^1,2^, Rivo Andry Rakotoarivelo^3^, Raphael Rakotozandrindrainy^4^, Rivo Solotiana Rakotomalala^5^, Sonya Ratefiarisoa^5^, Tahinamandranto Rasamoelina^6^, Jean-Marc Kutz^1,2^, Anna Jaeger^1^, Yannick Hoeppner^1^, Eva Lorenz^1,2^, Jürgen May^1,2 ,7^, Dewi Ismajani Puradiredja^1^, Daniela Fusco^1,2 *^

^1^ Department of Infectious Disease Epidemiology, Bernhard Nocht Institute for Tropical Medicine, Hamburg, Germany

^2^ German Center for Infection Research, Hamburg-Borstel-Lübeck-Riems, Germany

^3^ University Fianarantsoa, Fianarantsoa, Madagascar

^4^ University Antananarivo, Antananarivo, Madagascar

^5^ Centre Hospitalier Universitaire Androva, Mahajanga, Madagascar

^6^Centre Infectiologie Charles Mérieux, Antananarivo, Madagascar

^7^ Department of Tropical Medicine I, University Medical Center Hamburg-Eppendorf (UKE), Germany

*** Correspondence:**Daniela Fusco
fusco@bnitm.de

**Supplementary table 3:** Crude and adjusted prevalence ratios for FGS awareness among women (n=690), based on binary Poisson regression models with robust standard errors.

| **Group** | **N** | **CPR** | **APR** | **p-value*** |
| --- | --- | --- | --- | --- |
|  |  | (95% CI) | (95% CI) |  |
| **Location of Interview** |  |  |  |  |
| Healthcare | 116 | Reference | Reference | Reference |
| Community | 574 | 1.01 (0.58; 1.77) | 1.61 (0.87; 2.97) | 0.873 |
| **Age Group** |  |  |  |  |
| 18-25 | 247 | Reference | Reference | Reference |
| 26-35 | 224 | 1.63 (0.99; 2.68) | 1.55 (0.94; 2.56) | 0.089 |
| 36-45 | 117 | 1.38 (0.75; 2.54) | 1.56 (0.85; 2.88) | 0.155 |
| 46+ | 102 | 0.63 (0.27; 1.50) | 0.68 (0.28; 1.67) | 0.402 |
| **Urbanization** |  |  |  |  |
| Urban | 129 | Reference | Reference | Reference |
| Peri-Urban | 284 | 0.27 (0.16; 0.47) | 0.26 (0.15; 0.45) | <0.001 |
| Rural | 277 | 0.42 (0.26; 0.67) | 0.37 (0.22; 0.63) | <0.001 |
| **Education** |  |  |  |  |
| Primary education and less | 281 | Reference | Reference | Reference |
| Secondary education | 353 | 1.19 (0.75; 1.91) | 0.96 (0.58; 1.60) | 0.885 |
| University/ Vocational training | 56 | 2.51 (1.38; 4.58) | 2.00 (1.00; 4.00) | 0.05 |
| **Occupation** |  |  |  |  |
| Non-Farmer/ -Fisher | 261 | Reference | Reference | Reference |
| Farmer/ -Fisher | 429 | 0.71 (0.47; 1.08) | 1.00 (0.63; 1.58) | 0.997 |

** p-value multivariate model, Abbreviations: N= Number of participants, CPR= Crude prevalence ratio, APR= Adjusted prevalence ratio, CI= Confidence interval*
